# Macaque-tropic human immunodeficiency virus type 1: breaking out of the host restriction factors

**DOI:** 10.3389/fmicb.2013.00187

**Published:** 2013-07-09

**Authors:** Akatsuki Saito, Hirofumi Akari

**Affiliations:** ^1^Center for Human Evolution Modeling Research, Primate Research Institute, Kyoto UniversityInuyama, Japan; ^2^Japan Foundation for AIDS PreventionChiyoda-ku, Japan

**Keywords:** macaques, HIV-1, animal model, host factors, genetic background

## Abstract

Macaque monkeys serve as important animal models for understanding the pathogenesis of lentiviral infections. Since human immunodeficiency virus type 1 (HIV-1) hardly replicates in macaque cells, simian immunodeficiency virus (SIV) or chimeric viruses between HIV-1 and SIV (SHIV) have been used as challenge viruses in this research field. These viruses, however, are genetically distant from HIV-1. Therefore, in order to evaluate the efficacy of anti-HIV-1 drugs and vaccines in macaques, the development of a macaque-tropic HIV-1 (HIV-1mt) having the ability to replicate efficiently in macaques has long been desired. Recent studies have demonstrated that host restriction factors, such as APOBEC3 family and TRIM5, impose a strong barrier against HIV-1 replication in macaque cells. By evading these restriction factors, others and we have succeeded in developing an HIV-1mt that is able to replicate in macaques. In this review, we have attempted to shed light on the role of host factors that affect the susceptibility of macaques to HIV-1mt infection, especially by focusing on TRIM5-related factors.

## INTRODUCTION

It is estimated that about 2.5 million individuals per year get infected with human immunodeficiency virus type 1 (HIV-1), a causative agent of acquired immunodeficiency syndrome (AIDS; UNAIDS Global report 2012, ). To contain the disastrous epidemic, we need to consider effective approaches. For the pre-clinical evaluation of the anti-HIV-1 vaccines and therapy, it is necessary to have suitable animal models. Moreover, animal models would also aid for the understanding of the underlying mechanisms of HIV-1 pathogenicity. Since HIV-1 shows very narrow species specificity, being limited to human and apes, it has been quite challenging to develop an ideal animal model in which HIV-1 efficiently replicates and induces pathogenicity. Instead, many kinds of surrogate models developed as alternative strategy have provided us many important insights. In this decade, the molecular characterization of antiviral host restriction factors has dramatically progressed and shed light on the understanding of the viral specificity. These findings encouraged us to develop a novel non-human primate model for HIV-1 infection on the basis of a new concept (i.e., introduction of minimal modification to HIV-1 genome), by which the resultant virus would overcome a number of restriction factors. In this review, we summarize the history of the identification of the restriction factors and also discuss its impact and future direction on the development of HIV-1 animal models.

## HISTORY OF HIV-1 ANIMAL MODELS

### HIV-1 INFECTION IN SMALL ANIMALS

After the identification of HIV-1 as a causative agent of AIDS, many investigators sought to develop animal models for further research (reviewed in Gardner and Luciw, 1989). Although many efforts were performed in small animals, HIV-1 did not infect rodents, such as mice and rats, due to a number of restrictions, including the inability of HIV-1 Env to use the surface molecules in these animals as binding and entry receptors (Atchison et al., 1996) and the defect of murine cyclin T1 protein to associate with HIV-1 Tat (Kwak et al., 1999). Although rabbits were once expected to show susceptibility to HIV-1 infection (Filice et al., 1988; Kulaga et al., 1989), the reproducibility of this model remains to be elucidated (Reina et al., 1993; Speck et al., 1998; Tervo and Keppler, 2010). In an attempt to overcome the limitation in using these animals, several versions of humanized mice such as SCID-hu-PBL (severe combined immunodeficiency-human peripheral blood lymphocytes) mice (Mosier et al., 1988), Rag2^-^^/^^-^ γc^-^^/^^-^ mice (Traggiai et al., 2004), NOG (NOD/Shi-scid/IL-2Rγ null) mice (Ito et al., 2002), NSG (NOD scid gamma) mice (Shultz et al., 2005), and NOD/SCID-hu BLT mice (Melkus et al., 2006), have been generated (reviewed in Berges and Rowan, 2011). To generate this model, human immune cells were implanted into immunocompromised mice. After reconstitution of engrafted immune cells, HIV-1 replication in these animals was investigated. Generally, robust HIV-1 replication and loss of peripheral CD4^+^ T cells is observed in infected animals. Therefore, this model system would be useful for evaluation of drugs and neutralizing antibodies against HIV-1 (Denton et al., 2008). Moreover, this model provides important insight about the viral latency and the role of accessory genes *in vivo* (Denton et al., 2012; Marsden et al., 2012; Sato et al., 2012). However, none or weak immune response is observed in these animals. Moreover, this model requires special surgical skills and facilities to perform experiments.

### HIV-1 INFECTION IN NON-HUMAN PRIMATES

Differently from other pathogenic viruses for human such as measles and mumps, HIV-1 does not replicate in New World monkeys (NWMs) and Old World monkeys (OWMs). In cells from NWMs, such as squirrel monkey (*Saimiri sciureus*) and common marmoset (*Callithrix jacchus*), the cluster of differentiation 4 (CD4) and C-C chemokine receptor type 5 (CCR5) molecules function insufficiently as binding and entry receptors (LaBonte et al., 2002). On the other hand, in OWM cells, most HIV-1 enters target cells as efficiently as human cells. Interestingly, recent studies revealed that some subtypes of HIV-1 are unable to efficiently utilize macaque CD4 because of the difference in the C-terminus of the D1 domain of CD4 between human and OWMs, and therefore adaptive mutation was required for optimal efficiency (Humes and Overbaugh, 2011; Humes et al., 2012). After entering target cells, the subsequent steps of HIV-1 life cycle (i.e., uncoating and reverse transcription) are strongly abolished in OWM cells (Shibata et al., 1995; Hofmann et al., 1999). Although pigtailed macaque (*Macaca nemestrina*; hereafter denoted as PM) was once believed to be promising because of its higher susceptibility to HIV-1 infection as compared to other OWMs (Agy et al., 1992), the HIV-1 replication in those animals was weak and the trial of serial *in vivo* passage was shown to be unsuccessful (Agy et al., 1997). Among the animals examined for their susceptibility to HIV-1 infection, chimpanzees and gibbon apes were identified to have high susceptibility (Fultz et al., 1986; Lusso et al., 1988). In 1980s and 1990s, many chimpanzees were experimentally infected with HIV-1, including clinically isolated viruses and molecularly cloned viruses, resulting in a robust viral replication (Alter et al., 1984; Fultz et al., 1987, 1999; Nara et al., 1987; Prince et al., 1988). These experiments provided many important insights, including the roles of neutralizing antibody in protective immunity. While some of the infected chimpanzees experienced AIDS-related symptoms (Fultz et al., 1991; Novembre et al., 1997; O’Neil et al., 2000), most of them seemed not to develop apparent clinical symptoms (Gardner and Luciw, 1989; Johnson et al., 1993). Furthermore, there are many concerns about using chimpanzees, including ethical issues and their quite high rearing cost; therefore, researchers finally decided not to use this ape for HIV-1 research (Cohen, 2007). Therefore, the need for the development of other non-human primate models has been increasing.

### SIV INFECTION IN NON-HUMAN PRIMATES

As a surrogate model, simian immunodeficiency virus (SIV) infection in Asian macaques, such as rhesus macaque (*Macaca mulatta*; hereafter denoted as RM) and cynomolgus macaque (*Macaca fascicularis*; hereafter denoted as CM) has been developed. While SIV efficiently replicates in its natural host [e.g., sooty mangabey (*Cercocebus atys*; hereafter denoted as SM) for SIVsm and African green monkey (*Chlorocebus sabaeus*; hereafter denoted as AGM) for SIVagm, respectively; Ohta et al., 1988; Kraus et al., 1989], infected animals generally do not develop immunodeficiency, unlike the course of HIV-1 infection in humans. In the 1980s, accidental transmission of SIVsm to RMs caused a lethal disease, and the symptoms were quite similar to those seen in AIDS patients (Daniel et al., 1985; Letvin et al., 1985). Thereafter, the pathogenic virus was molecularly cloned as SIVmac (Naidu et al., 1988; Kestler et al., 1990). The combination of SIVmac and RMs has been broadly utilized as a surrogate model for HIV-1 infection because of its similarity in the genome structure and pathogenicity. Specifically, this model dramatically advanced our understanding in terms of the functional roles of the viral accessory genes *in vivo *(Kestler et al., 1991; Gibbs et al., 1995; Hirsch et al., 1998). Moreover, this model provided the important finding that the acquired protective immunity induced by live-attenuated vaccines was effective against homologous and heterologous SIV challenges (Daniel et al., 1992; Wyand et al., 1996, 1999).

### INFECTION OF CHIMERIC VIRUS BETWEEN HIV-1 AND SIV IN NON-HUMAN PRIMATES

Accumulating evidence has demonstrated the inability of intact HIV-1 to replicate in OWM cells. Then, what kind of viral components in HIV-1 and SIV determine their host tropism? In an effort to answer this profound question, many researchers constructed chimeric viruses between HIV-1 and SIV and analyzed their viral replication in human and OWM cells. It was shown that chimeric viruses containing HIV-1-derived *gag* and/or *vif* were unable to replicate in macaque cells and that a chimeric virus encoding HIV-1-derived *env *on the SIVmac backbone was able to replicate in primary OWM cells (Shibata et al., 1991; Shibata and Adachi, 1992), indicating that the step of entry was not the determinant for the species specificity of HIV-1. As a consequence of vigorous investigation, Shibata et al. finally succeeded to construct a prototypic simian–human immunodeficiency virus (hereafter denoted as SHIV) clone that encodes HIV-1-derived *tat*, *rev*, *vpu*, and *env* genes on the SIVmac239 backbone (Shibata et al., 1991; Shibata and Adachi, 1992). This SHIV clone was shown to efficiently replicate in primary macaque cells. Thereafter, many groups developed several versions of SHIV. Of note, by serial passaging of apathogenic SHIV-89.6 in monkeys, Reimann et al. (1996)**successfully obtained a highly pathogenic virus (SHIV-89.6P) that caused rapid and complete depletion of peripheral CD4^+^ T cells, leading to simian AIDS. These chimeric viruses not only enabled us to evaluate the efficacy of antiviral immunity against HIV-1 Env but also supplied us important insights on what kind of SIVmac-derived genes are necessary to replicate in macaque cells. This SHIV model became a huge breakthrough for HIV-1 investigators; by using SHIV, the mechanism and efficacy of passive immunization (Shibata et al., 1999; Baba et al., 2000; Nishimura et al., 2002) as well as vaccine candidates (Igarashi et al., 1997; Letvin et al., 1997; Cafaro et al., 1999) were vigorously investigated. Incidentally, the lower sequence homology in RT between SIV and HIV-1 limited this model for the evaluation of antiretroviral drugs especially against RT. To overcome this limitation, RT-SHIV, which encodes HIV-1 RT in the place of SIVmac RT, was developed and used for the assessment of RT inhibitors (Uberla et al., 1995; Ambrose et al., 2004; North et al., 2005). SHIV carrying HIV-1 integrase (IN) in addition to RT was also constructed (Akiyama et al., 2008). These efforts have dramatically advanced the basic research related to HIV-1. However, since these viruses were constructed on the basis of SIVmac backbone, SHIVs are still far from HIV-1. Moreover, some pathogenic SHIV clones, such as SHIV-89.6P, show quite different phenotypes in macaques, unlike those in HIV-1 infection of humans and SIVmac infection of macaques (Feinberg and Moore, 2002). First, these SHIVs induced abnormally rapid, profound, and irreversible loss of CD4^+^ T cells in macaques, differently from the gradual decline of CD4^+^ T cells observed in most HIV-1-infected patients (McCune, 2001). Second, these SHIVs were somehow highly susceptible to neutralizing antibodies, while most HIV-1 isolates and pathogenic SIVs were resistant to neutralization. Therefore, earlier vaccine studies using SHIV as a challenge virus succeeded in controlling viral replication by immunization with vaccine candidates (Amara et al., 2001; Barouch et al., 2001). Notably, these outcomes were frequently observed in experiments with SHIV using C-X-C chemokine receptor type 4 (CXCR4; X4-tropic virus), or SHIV using both CXCR4 and CCR5 as co-receptors (dual-tropic virus). Since HIV-1 in human population usually uses CCR5 as a co-receptor during transmission (Schuitemaker et al., 1992), it will be straightforward to develop an R5-tropic SHIV in order to reproduce the transmission, latency, and pathogenicity of HIV-1 in macaques. In fact, R5-tropic SHIVs were recently constructed and their phenotype seemed different from those of X4-tropic SHIVs and dual-tropic SHIVs. It is thought that X4-tropic SHIV selectively infects CXCR4^+^ naive CD4^+^ T cells that are enriched in secondary lymph nodes, while most SIV and R5-tropic SHIV mainly target CCR5^+^ memory CD4^+^ T cells in extra-lymphoid immune effector sites such as gut, lung and genital tract, explaining the divergent clinical sequel (Harouse, 1999; Nishimura et al., 2004; Ho et al., 2005). Especially, mucosal infection with R5-tropic SHIV would be a promising tool for investigating protection and transmission of immunodeficiency viruses (Matsuda et al., 2010; Bomsel et al., 2011; Gautam et al., 2012; Moldt et al., 2012).

In spite of the usefulness of these SHIVs in experiments targeting HIV-1 *env*, the low similarity in other genes, especially *gag* and *pol*, still limits the use of this virus as a challenge virus. Since cytotoxic T lymphocyte (CTL) response against Gag protein is thought to play a central role in controlling viral replication (Kiepiela et al., 2007), the absence of HIV-1-derived *gag* in current SHIV hampers evaluation of vaccine candidate against HIV-1 Gag. To solve this problem, we need to proceed to construct more relevant animal models of HIV-1. In this decade, our knowledge about host factors that form species barrier against HIV-1 has dramatically increased. This knowledge would permit us to develop an HIV-1 clone having the potential to replicate in macaques. Many efforts to develop a more feasible model were made by several groups as described below. Here, we summarize the role of anti-HIV-1 restriction factors in macaque cells and the viral antagonists against these factors.

## INTRINSIC HOST FACTORS

### APOBEC3 FAMILY

It has long been observed that the infectivity of *vif* gene-deficient HIV-1 in certain T cell lines such as H9 and CEM, as well as primary lymphocytes, was strongly decreased (Gabuzda et al., 1992; Sakai et al., 1993; Tervo and Keppler, 2010). Virions produced from these restrictive cells have less infectivity as compared to the wild-type virus. Many efforts were made to identify a cellular factor that conferred this restrictive activity. In particular, the fact that heterokaryons between permissive and restrictive cells suppressed the infectivity of the *vif*-deficient HIV-1 clearly suggested the existence of a potent endogenous inhibitor of HIV-1 replication in restrictive cells (Madani and Kabat, 1998; Simon et al., 1998). Finally, in 2002, the apolipoprotein B mRNA editing enzyme catalytic polypeptide 3 G (APOBEC3G; hereafter denoted as A3G) was identified as a novel host restriction factor in human cells (Sheehy et al., 2002). A3G is expressed in various tissues including testis, ovary, spleen, and peripheral blood mononuclear cells (PBMCs; Jarmuz et al., 2002). Since A3G is a member of the cytidine deaminase enzyme, the *vif*-deficient virus contains many G-to-A mutations in its minus-strand genome, leading to disruption of infectivity. Moreover, the fact that deamination-deficient mutant A3G can still inhibit *vif*-deficient HIV-1 implied that A3G exerts its antiviral activity with deamination-dependent and deamination-independent fashion (Newman et al., 2005). In order to counteract the A3G-mediated restriction, HIV-1 has equipped its genome with *vif* gene and the resultant protein, Vif, efficiently inhibits A3G incorporation into progeny virions by inducing proteasome-dependent degradation of A3G (Conticello et al., 2003; Kao et al., 2003; Mehle et al., 2004). Recently, it was reported that core-binding factor beta (CBFβ), a transcription regulator through RUNX binding, was required for HIV-1 Vif to degrade A3G (Hultquist et al., 2012; Jager et al., 2012). SIVmac Vif similarly recruits CBFβ in order to neutralize the RM A3G (Hultquist et al., 2012; Jager et al., 2012). It was also proposed that HIV-1 Vif suppresses human A3G activity by inhibiting the translation of A3G (Mercenne et al., 2010). Although the human genome encodes other six A3 members (A3A, B, C, DE, F, and H) in addition to A3G, the precise antiviral activity of the A3 proteins remains to be elucidated. Human A3F was also reported to have anti-HIV-1 activity and susceptibility to HIV-1 Vif (Liddament et al., 2004; Wiegand et al., 2004; Zheng et al., 2004). In contrast, Miyagi et al. (2010) suggested that the antiviral activity of endogenous level of human A3F was negligible as compared to the activity of A3G. It is known that human A3DE and A3F, in addition to A3G, are also sensitive to counteraction by HIV-1 Vif (Goila-Gaur and Strebel, 2008). As seen in humans, the RM genome also encodes seven A3 members (Schmitt et al., 2011). Virgen and Hatziioannou (2007) investigated the susceptibility of HIV-1 to each RM A3 family member and showed that A3B, A3F, A3G, and A3H had the ability to restrict HIV-1 and were resistant to HIV-1 Vif activity. It should be noted that Vif-A3G interaction shows species specificity (Mariani et al., 2003). HIV-1 Vif is able to counteract A3G from humans but not from RM and AGM (Zennou and Bieniasz, 2006; Virgen and Hatziioannou, 2007). Conversely, SIVagm Vif is effective against A3G from AGM and RM, but unable to antagonize A3G from human and chimpanzee (Mariani et al., 2003). SIVmac Vif efficiently counteracts A3G from human, chimpanzee, AGM, and RM (Mariani et al., 2003). Are there any polymorphisms in the *A3* family? In case of humans, a polymorphism in* A3B* deletion was reported (Kidd et al., 2007). In RMs, a polymorphism in A3DE was observed and was reported to affect the antiviral activity (Virgen and Hatziioannou, 2007). How can we obtain HIV-1 with the ability to overcome macaque A3s? Many efforts have been made to evade from the restriction by the macaque A3 family. Schrofelbauer et al. (2006)**showed that mutations of HIV-1 Vif at positions 14–17 from DRMR into SEMQ allowed HIV-1 Vif interaction with A3G from RM. However, this HIV-1 Vif harboring SEMQ remained susceptible to A3B, A3F, and A3H from RM (Virgen and Hatziioannou, 2007), suggesting that the introduction of this sequence in HIV-1 Vif was not sufficient for evading from A3s other than A3G. Besides, since the replication of HIV-1 in OWM cells was suppressed, at least at two steps (early and late stages of HIV-1 lifecycle), it is reasonable to speculate that just a modification of *vif* is insufficient for HIV-1 to overcome the restriction in various OWM cells.

### BONE MARROW STROMAL ANTIGEN 2

It had been observed that the production of *vpu*-deficient HIV-1 in certain cell lines was severely diminished (Klimkait et al., 1990; Sakai et al., 1995). Specifically, while permissive cells, such as HEK293T and HT1080 cells, allowed comparative levels of virion production, non-permissive cells, such as Jurkat and HeLa cells, decreased the amount of virion production in the absence of *vpu*. It was also reported that interferon (IFN) treatment led to phenotype switch from permissive to non-permissive (Neil et al., 2007). Thus, the existence of unknown IFN-inducible, Vpu-sensitive cellular factors, was predicted. In 2008, bone marrow stromal antigen 2 (BST-2), also known as tetherin, CD317, and HM1.24, was identified by two independent groups (Neil et al., 2008; Van Damme et al., 2008). BST-2 is a type 2 integral membrane protein, with the N-terminus located in the cytoplasm, one membrane-spanning domain, and a C-terminus modified by the addition of a glycosyl-phosphatidylinositol (GPI) anchor (Kupzig et al., 2003). Erikson et al. (2011) analyzed the expression profile of BST-2 *in vivo* and demonstrated that BST-2 was expressed in various tissues, especially spleen and alimentary system. They also showed that among PBMCs, monocytes express high levels of BST-2 as compared to T and B cells. Furthermore, like tripartite motif-containing protein 5 (TRIM5α), hominid BST-2, but not other primate BST-2, has been recently reported to function as an innate sensor, leading to the transforming growth factor β activated kinase-1 (TAK1)-dependent activation of NFκB and subsequent production of pro-inflammatory cytokines (Galao et al., 2012). Cocka and Bates (2012) recently showed that human *BST-2* gene expressed alternative splice isoforms that led to different antiviral activity as well as sensing activity from the wild-type one. To overcome BST-2-mediated restriction, HIV-1 downregulates BST-2 from the cell surface by expressing Vpu protein, a viral protein absent in most of the SIVsm/HIV-2 lineage (Neil et al., 2008; Van Damme et al., 2008). On the other hand, HIV-2 utilizes Env protein as an antagonist for human BST-2 (Le Tortorec and Neil, 2009). In the case of SIVmac, Nef protein confers the ability to overcome BST-2-mediated restriction in RM cells (Jia et al., 2009; Sauter et al., 2009; Zhang et al., 2009). It is also reported that Env protein of SIVtan [SIV from Tantalus monkeys (*Chlorocebus tantalus*)] was effective against BST-2 from human and RM (Gupta et al., 2009). It should be noted that the antagonistic activity of these viral proteins against BST-2 is thought to function in a species-specific manner. While Vpu from the HIV-1 group M is able to counteract human and chimpanzee BST-2, most of these Vpus are ineffective against BST-2 from RM and AGM (McNatt et al., 2009; Sauter et al., 2009). In contrast, Nef from SIVmac is effective for BST-2 from RM and SM but ineffective for BST-2 from human (Jia et al., 2009). This characteristic resistance of human BST-2 to SIV Nef was proven to have an association with the deletion in human BST-2 of 5 amino acid residues, to which SIV Nef binds (Jia et al., 2009; Zhang et al., 2009). Although most SIVsm/HIV-2 lineage does not encode *vpu* gene, SIVcpz, SIVgor [SIV from gorillas (*Gorilla gorilla gorilla*)], SIVgsn [SIV from greater spot-nosed guenons (*Cercopithecus nictitans*)], SIVmon [SIV from mona monkeys (*Cercopithecus mona*)], SIVmus [SIV from moustached monkey (*Cercopithecus cephus*)], and SIVden [SIV from Dent’s mona monkey (*Cercopithecus denti*)] were shown to harbor the *vpu* gene (Courgnaud et al., 2003; Dazza et al., 2005). Recently, Sauter et al. (2009)**demonstrated that Vpus from SIVgsn and SIVden potently counteracted the BST-2 from RM. Moreover, Shingai et al. (2011) showed that Vpu from SHIV_DH12_ potently counteracted BST-2 from RM. It is therefore possible that the exchange of present HIV-1_NL4-3_-derived-Vpu with these Vpus might lead to efficient evasion from the BST-2-mediated restriction in macaque cells. It was reported that a *nef*-deleted SIVmac239 inoculated to RM became pathogenic after *in vivo* passage (Alexander et al., 2003; Serra-Moreno et al., 2011). Serra-Moreno et al. (2011) showed that the *nef*-deleted SIVmac239 gained the ability to antagonize BST-2 by utilizing its Env gp41 as a consequence of adaptive mutations in the *env* gene. In addition, Vpu from the less pathogenic HIV-1 group O was reported to lose anti-BST-2 activity (Sauter et al., 2009). It was shown that SHIV_DH12_ lacking intact Vpu inefficiently replicated *in vivo* as compared to the wild-type virus (Shingai et al., 2011). These findings indicate the importance of evasion from BST-2-mediated restriction for lentiviral pathogenesis *in vivo*. Although detailed genetic information is limited, the *BST-2* gene is reported to be polymorphic at least in RM (McNatt et al., 2009). Therefore, when using macaques for HIV-1 research, we should also appreciate the polymorphisms in *BST-2* gene.

### SAMHD1

It has long been observed that HIV-1 replication in myeloid linage cells, such as macrophages and dendritic cells (DCs) was impaired and the expression of HIV-2/SIV Vpx *in trans* was shown to rescue this inhibition (Goujon et al., 2007, 2008; Kaushik et al., 2009). The sterile alpha motif (SAM) and histidine/aspartic acid (HD) domain containing protein 1 (SAMHD1) was identified as an HIV-1 restriction factor in myeloid cells that were degraded by the HIV-2/SIV Vpx protein (Hrecka et al., 2011; Laguette et al., 2011). SAMHD1 was reported to restrict HIV-1 replication in resting CD4^+^ T cells as well (Baldauf et al., 2012; Descours et al., 2012). Historically, SAMHD1 is shown to be associated with the Aicardi–Goutières autoimmune-mediated neurodevelopmental syndrome. Patients having a mutation in *SAMHD1* gene would have symptoms of abnormal immune activation likely due to the excessive production of IFNα (Crow and Rehwinkel, 2009; Rice et al., 2009). Since SAMHD1 functions as a deoxyguanosine triphosphate (dGTP)-regulated deoxynucleoside triphosphate (dNTP) triphosphohydrolase (Powell et al., 2011), it exerts its anti-HIV-1 activity via the depletion of dNTP pools in virus-infected cells, leading to the inhibition of the reverse transcription (Lahouassa et al., 2012). The fact that SAMHD1-deficient CD14^+^ monocytes efficiently permit HIV-1 replication supports this notion (Berger et al., 2011). It is noteworthy that SAMHD1 exerts its antiviral activity against various retroviruses ranging from alpha, beta and gamma retrovirus, except for prototype foamy virus and Human T cell leukemia virus type I (HTLV-1; Gramberg et al., 2013). As described above, the SAMHD1-mediated restriction would be counteracted by HIV-2/SIV Vpx. Hofmann et al. (2012) showed that Vpx recruits SAMHD1 to a cullin4 A-RING E3 ubiquitin ligase, leading to proteasomal degradation. The importance of Vpx *in vivo* was based on the fact that the replication of *vpx*-deleted SIV in monkeys was significantly weaker than that in wild-type SIV (Gibbs et al., 1995; Hirsch et al., 1998; Belshan et al., 2012). However, *vpx*-deleted SIV still had the ability to induce simian AIDS in macaques, suggesting a limited role of SAMHD1-mediated restriction in SIV pathogenesis (Gibbs et al., 1995). It is of note that while HIV-2 as well as most of SIV linage such as SIVmac encodes *vpx*, HIV-1 as well as some SIV lineage such as SIVcpz and SIVgor does not encode *vpx* in its genome. Similar to the relationship between A3G and Vif, SAMHD1 is also antagonized by viral proteins in a species-specific manner. For instance, Vpxs from SIVmac and SIVsm are effective against SAMHD1 from human, OWMs, and NWMs (Laguette et al., 2012), while those from SIVrcm [SIV from red-capped mangabey (*Cercocebus torquatus*)] or SIVmnd [SIV from mandrill (*Mandrillus sphinx*)] are effective against SAMHD1 from OWMs and NWMs but not from humans (Lim et al., 2012). Lim et al. (2012) also found that Vpr from some SIV lineage, such as SIVdeb [SIV from De Brazza’s monkey (*Cercopithecus neglectus*)], SIVmus, and a part of SIVagm (SIV from AGM), has the potency of degrading SAMHD1 from RM and AGM. It would be of great interest to introduce these *vpr*s into HIV-1mt and examine whether this modification would enhance the viral replication in myeloid linage cells from macaques.

### TRIM5

It was demonstrated that the replication of HIV-1 in OWMs cells was severely abolished before reverse transcription (Besnier et al., 2002; Cowan et al., 2002; Munk et al., 2002). An experiment using interspecies heterokaryons between OWM and human cells suggested the existence of an inhibitory factor in OWM cells (Munk et al., 2002). Stremlau et al. (2004) by screening the RM cDNA library, successfully identified TRIM5α as a restriction factor in RM cells that confer permissive cells resistance to HIV-1 infection. They also demonstrated that RM TRIM5α, but not human TRIM5α, could restrict HIV-1 infection. On the other hand, human TRIM5α potently restricts the N-tropic murine leukemia virus (N-MLV) as well as the equine infectious anemia virus (EIAV) but not B-tropic murine leukemia virus (B-MLV; Hatziioannou et al., 2004; Keckesova et al., 2004; Perron et al., 2004; Yap et al., 2004), indicating the importance of TRIM5α as a host factor restricting the cross-species transmission of retroviruses. TRIM5α is ubiquitously expressed and consists of a RING domain, a B-box domain, a coiled coil domain, and a PRYSPRY (B30.2) domain (Reymond et al., 2001). The characteristic PRYSPRY domain recognizes the capsid of incoming retroviruses, leading to the restriction of the infection at the post-entry step. This domain is also responsible for the species-specific function of TRIM5α (Nakayama and Shioda, 2010). It was shown that TRIM5α was IFN-inducible and that IFN treatment of cells led to the augmentation of antiviral activity (Asaoka et al., 2005; Sakuma et al., 2007). An additional role of TRIM5α as a pattern recognition receptor was recently identified (Pertel et al., 2011). TRIM5α binds to the incoming viral capsid and then activates its E3 ligase activity, together with the UBC13–UEV1A enzyme complex, resulting in the synthesis of free ubiquitin chains. The chains stimulate TAK1 phosphorylation and the expression of NF-κB (nuclear factor kappa-light-chain-enhancer of activated B cells)- and MPK (mitogen-activated protein kinase)-responsive genes, leading to an antiviral state (De Silva and Wu, 2011). Among the restriction factors discussed here, *TRIM5* gene might be most polymorphic in primates. At what degree does this polymorphism in *TRIM5* gene affect the susceptibility to retroviral infection? A length polymorphism in *TRIM5α*, in which the TFP residues from position 339 to 341 of TRIM5α were replaced with a single glutamine (Q), was identified in some RM individuals (Newman et al., 2006). This TFP/Q polymorphism affects the anti-lentiviral activity of RM TRIM5α against SIVsmE543-3 and SIVsmE041 but not against SIVmac (Kirmaier et al., 2010). Similarly, this polymorphism in RM *TRIM5α* is associated with the different antiviral activity against HIV-2 (Kono et al., 2008).

Although most cell lines from NWMs were susceptible to VSV-G pseudotyped HIV-1, cell lines from owl monkey (*Aotus trivirgatus*) exceptionally showed high resistance to infection by HIV-1 (Hofmann et al., 1999). As the reason for this discrepancy, Sayah et al. (2004) successfully identified TRIM5-Cyclophilin A (CypA) chimeric protein (referred to as TRIMCyp) in owl monkey, which was derived from LINE-1-mediated retrotransposition of CypA cDNA into the region between *TRIM5* exons 7 and 8. In the case of OWMs, the higher susceptibility of PM to HIV-1 infection was, at least in part, explained by the fact that PM exclusively have the *TRIMCyp* genotype instead of TRIM5α (Liao et al., 2007; Brennan et al., 2008; Virgen et al., 2008). Differently from owl monkey TRIMCyp, the *TRIMCyp* of PM was a consequence of a retrotransposition of the *CypA* sequence in the 3′ untranslated region (UTR) of the *TRIM5* gene, together with a single nucleotide polymorphism (SNP) at the exon 7 splice acceptor site. This SNP at the splice acceptor site leads to skipping exons 7 and 8 encoding the PRYSPRY domain and splicing to the inserted *CypA* gene. In addition to PM, it is reported so far that RM and CM also possess *TRIMCyp* in their genome (Brennan et al., 2008; Newman et al., 2008; Wilson et al., 2008). Interestingly, RM has geographic deviation in the frequency of *TRIMCyp*, depending on the country of origin (Wilson et al., 2008). It is reported that Indian RM possessed *TRIMCyp* more frequently than Chinese RM (Wilson et al., 2008; De Groot et al., 2011). We recently reported that CM also showed divergent frequency of *TRIMCyp* depending on their country of origin (Saito et al., 2012b). The frequency of *TRIMCyp* in Filipino CM was significantly higher than that in Malaysian and Indonesian CM. We demonstrated that wild-caught CM also had a geographic deviation in the frequency of *TRIMCyp* as seen in captive CM (Saito et al., 2012a). Consistently, Dietrich et al. (2011) reported that the frequency of *TRIMCyp* in Filipino CM was higher than those in Indonesian and Indochina CM. It was shown that Mauritian CM, a population thought to be derived from Indonesian CM, seemed not to possess *TRIMCyp*, probably due to the founder effects at the time of introduction by human (Dietrich et al., 2011; Berry et al., 2012). Since TRIM5α is expected to act as homomultimer (Mische et al., 2005; Perez-Caballero et al., 2005), heterologous expression of *TRIM5α* in combination with *TRIM5* isoforms other than TRIM5α reportedly led to a dominant negative effect on the TRIM5α antiviral activity (Berthoux et al., 2005; Maegawa et al., 2008). Interestingly, it was reported that RM heterozygous for *TRIM5α* and *TRIMCyp* showed higher resistance to repeated intrarectal challenge of SIVsmE660 as compared to RM homozygous for *TRIM5α* or *TRIMCyp* (Reynolds et al., 2011). Since RM TRIMCyp could restrict SIVsm but not SIVmac (Kirmaier et al., 2010), it is reasonable to assume that the combination of *TRIM5α* and *TRIMCyp* may function more efficiently as antiviral factors against SIVsm. We will further discuss the impact of *TRIM5* polymorphism on the viral replication in the latter chapter. In summary, since *TRIM5* genotype would greatly influence the susceptibility to lentiviruses, the correlation between polymorphism of *TRIM5* gene in macaques and outcomes should be carefully evaluated.

### UNIDENTIFIED RESTRICTION FACTORS

Viral infection usually stimulates cellular factors through pattern recognition receptors, such as Toll-like receptor (TLRs) and RIG-I-like receptors, expressed on many type of cells, leading to the induction of IFN production (Bowie and Unterholzner, 2008). In particular, type I IFN, which include IFN-α and IFN-β, puts a switch on the IFN-stimulated gene 15 (ISG15), leading to a cascade of antiviral status (Zhao et al., 2013). The expression levels of the restriction factors described above are reported to increase via IFN stimulation (Asaoka et al., 2005; Tanaka et al., 2006; Neil et al., 2007; Sakuma et al., 2007). Lately, Bitzegeio et al. (2013) have demonstrated that HIV-1-based chimeric viruses, engineered to overcome SAMHD1 or BST-2 as well as A3 and TRIM5 from PM, are still severely restricted in IFN-treated PM PBMCs. They have also demonstrated that the replication of SIVmac in IFN-treated human PBMCs is greatly suppressed, and *vice versa*. This finding strongly suggests the existence of unidentified, IFN-inducible restriction factors in each species. Therefore, it is also necessary to continue exploring such unidentified cellular factors.

## CONSTRUCTION OF MACAQUE-TROPIC HIV-1

**FIGURE 1:**
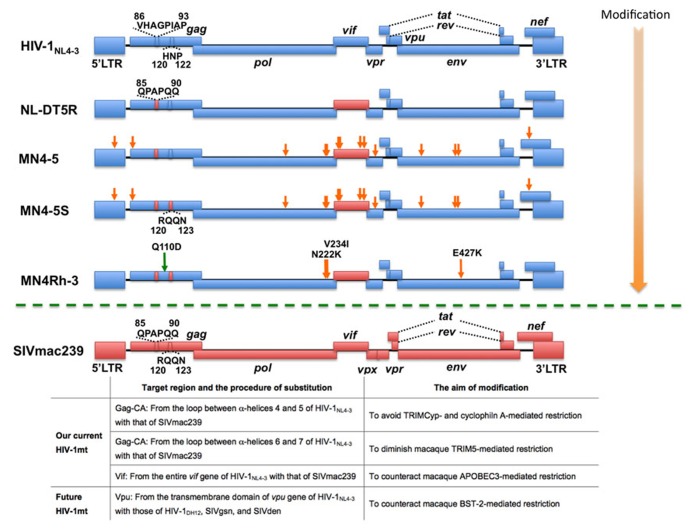
**Structure of HIV-1mt clones.** Blue boxes denote HIV-1_NL4-3_-derived sequences and red boxes denote SIVmac239- derived sequences, respectively. Orange arrows show adaptive mutations that enhance viral growth potential in macaque T cell lines. The green arrow indicates the CA-Q110D mutation. (Bottom) Summary of the modifications in our current and future HIV-1mts in order to evade from restriction factors in macaque cells.

**FIGURE 2:**
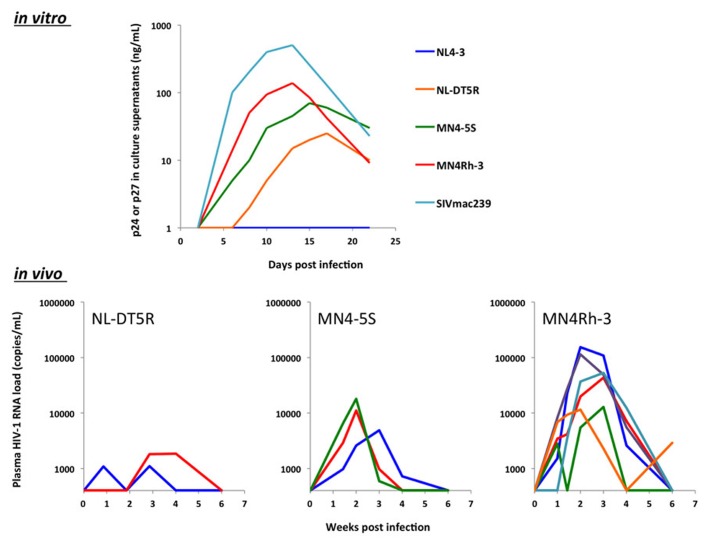
**Serial modifications of the viral genome lead to an enhanced viral replication* in vitro* and *in vivo*.** (Top) CD8^+^ T cell-depleted peripheral mononuclear blood cells from CMs homozygous for *TRIMCyp* were infected with each HIV-1mt. The representative result of viral replication kinetics was shown. (Bottom) CM having *TRIMCyp* were infected with each HIV-1mt intravenously. Plasma viral RNA loads in each monkey are shown.

In virtue of the detailed understanding of the molecular relationship between antiviral host factors and viral antagonists (summarized in **Tables 1** and **2**), it became possible to create a macaque-tropic HIV-1 (HIV-1mt) with the ability to replicate in OWM cells. In 2006, two independent groups succeeded in the construction of an HIV-1mt that contains partial SIV-derived sequences on the HIV-1_NL4-3_ backbone. Hatziioannou et al. (2006) constructed HIV-1mt that contains the entire Gag-CA and *vif* from SIVmac in order to evade from TRIM5α- and A3G-mediated restriction, respectively. This HIV-1mt, which contains approximately 88% of HIV-1-derived sequence, was shown to efficiently replicate in RM PBLs. In parallel with that study, Kamada et al. (2006)**constructed HIV-1mt named NL-DT5R in which the sequence of CypA binding loop [the loops of α-helices 4 and 5 (L4/5)] in Gag-CA and entire *vif *gene were replaced with those from SIVmac239. NL-DT5R, in which approximately 93% of its sequence was derived from HIV-1, was shown to replicate in a CM T cell line (HSC-F cells) as well as CD8^+^ cell-depleted PM PBMCs but hardly in CD8^+^ cell-depleted RM PBMCs. Subsequently, Igarashi et al. (2007) investigated the replication capability of NL-DT5R in PM and found that this prototypic HIV-1mt was able to induce acute viremia up to around 1 × 10^4^ copies/mL. Thereafter, in order to enhance the viral replication, we further modified the sequence of NL-DT5R-based HIV-1mt by 2 different approaches. First, we performed a long-term adaptation experiment in CM T cell lines to induce adaptive mutation in its genome. As a consequence of adaptation, several nucleotide substitutions were identified (see **Figure 1**, orange arrows in MN4-5 and MN4-5S). The functional significance of each mutation was molecularly evaluated (Nomaguchi et al., 2013a). Second, we introduced the α-helices 6 and 7 (L6/7) in addition to L4/5 of Gag-CA into MN4-5, resulting in MN4-5S. As shown in **Figure 2**, this substitution enhanced the viral replication *in vitro *(Kuroishi et al., 2009) and *in vivo *(Saito et al., 2011). We next constructed a new HIV-1mt named MN4Rh-3 carrying the Q110D substitution in Gag-CA. This HIV-1mt exhibited further enhanced growth property specifically in macaque cells but impaired replication in human cells (Nomaguchi et al., 2013b). We also examined the replicative property of MN4Rh-3 in CM (Saito et al., 2013). In accordance with* in vitro* data (Nomaguchi et al., 2013b), MN4Rh-3 induced higher viremia on average up to 50 times as compared to MN4-5S (**Figure 2**). Notably, *TRIMCyp* homozygotes were highly permissive to MN4Rh-3 infection, while the replication of MN4Rh-3 in *TRIM5* homozygotes was strongly suppressed. We also observed that CM heterologous for *TRIM5α* and *TRIMCyp* showed similar anti-HIV-1 activity with *TRIMCyp *homozygotes (Saito et al., 2013). These findings indicated that MN4Rh-3 enhanced the replicative capability in CM having *TRIMCyp*, but was still unable to overcome TRIM5α-mediated restriction. It should be noted that the sequence of most *TRIMCyp* encoded in CM are different from those in RM and PM. It was once thought that CM exclusively possessed TRIMCyp in which the amino acid residues at positions 369 (Cyp66) and 446 (Cyp143) were aspartic acid (D) and lysine (K) [denoted as the TRIMCyp (DK)], respectively, while PM and RM had TRIMCyp in which the amino acids at the corresponding positions were asparagine (N) and glutamic acid (E) [denoted as the TRIMCyp (NE); Brennan et al., 2008; Ylinen et al., 2010], respectively. However, others and we recently revealed that CM possessed TRIMCyp (NE) as well as TRIMCyp (DK) in spite of the low frequency of TRIMCyp (NE) haplotype in CM population (Dietrich et al., 2011; Saito et al., 2012a, b). Strikingly, others and we reported that TRIMCyp (DK) and TRIMCyp (NE) exhibit different anti-lentiviral activity. It is well established that the TRIMCyp (DK) efficiently restricts HIV-1 but weakly restricts HIV-2 (Saito et al., 2012b). On the other hand, the TRIMCyp (NE) fails to restrict HIV-1 but efficiently restrict HIV-2 (Wilson et al., 2008). It was also shown that both haplotypes hardly restricted SIVmac239 replication. These results indicate that the sequence variations in CypA greatly affect the spectrum of their anti-HIV-1 activity. However, how does TRIMCyp (DK) exert its anti-HIV-1 activity? Actually, TRIMCyp (DK) is expected to bind the L4/5 in Gag-CA. Moreover, the treatment of the target cells with cyclosporin A, an inhibitor against CypA, or the introduction of amino acid changes in this loop of the viral genome relieved the inhibitory effect by TRIMCyp (DK; Ylinen et al., 2010). Therefore, when we use CM homozygous for TRIMCyp (DK), it is necessary to modify the loop in order to evade restriction. In fact, we have used HIV-1mts in which the L4/5 in Gag-CA were replaced with the corresponding sequence of SIVmac239 (Kamada et al., 2006). In contrast, those research groups that used PM did not need to modify this region. Hatziioannou et al. (2009) have successfully constructed an HIV-1mt that induced persistent viremia in PM with modification of only *vif* and *env *gene. Similarly, Thippeshappa et al. (2011)**also constructed an HIV-1mt named HSIV-*vif* that encoded *vif* gene from pathogenic PM-adapted SIVmne027. This HSIV-*vif *was shown to persistently replicate in PM but was unable to induce pathogenicity in animals. Overall, further understanding of the host–virus relationship would permit us to construct pathogenic HIV-1mt in future studies.

**Table 1:** Antiviral host factors and antagonism by lentiviral proteins.

Antiviral host factors	Antagonized by	NOT antagonized by
Human APOBEC3G	HIV-1 Vif	SIVagm Vif
	SIVmac Vif	
RM APOBEC3G	SIVmac Vif	HIV-1 Vif
	SIVagm Vif	
Human BST-2	HIV-1 Vpu	HIV-1 Nef
	HIV-2 Env	SIVmac Nef
RM BST-2	SIVgsn Vpu	HIV-1 Vpu*
	SIVden Vpu	SIVmac Nef
	SIVmac Nef	
Human SAMHD1	SIVdeb Vpr	HIV-1 Vpr
	SIVmus Vpr	SIVmac Vpr
	SIVmac Vpx	SIVrcm Vpx
	HIV-2 Vpx	SIVmnd Vpx
RM SAMHD1	SIVdeb Vpr	HIV-1 Vpr
	SIVmus Vpr	SIVmac Vpr
	SIVagm Vpr	SIVrcm Vpr
	SIVmac Vpx	
	HIV-2 Vpx**	
	SIVrcm Vpx	
	SIVmnd Vpx	

Summary of findings about APOBEC3G–Vif interaction (Sheehy et al., 2002; Kao et al., 2003; Mariani et al., 2003; Zennou and Bieniasz, 2006; Virgen and Hatziioannou, 2007), BST-2–Vpu, Nef, and Env interaction (Neil et al., 2008; Van Damme et al., 2008; Jia et al., 2009; Le Tortorec and Neil, 2009; Sauter et al., 2009; Zhang et al., 2009; Serra-Moreno et al., 2011), and SAMHD1-Vpx and Vpr interaction (Hrecka et al., 2011; Laguette et al., 2011, 2012; Lim et al., 2012). *Vpus from some HIV-1 strains such as HIV-1_DH12_ are able to antagonize RM BST-2. **Vpxs from some HIV-2 strains are ineffective in antagonizing RM SAMHD1.

**Table 2:** Species-specific restriction of lentiviruses by primate TRIM5 proteins.

*TRIM5* alleles	Restrictive against:	
	HIV-1	HIV-1mt MN4Rh-3	SIVmac239
Human TRIM5α	-	-	-
RM TRIM5α (TFP)	+	+	-
RM TRIM5α (Q)	+	+	-
CM TRIM5α	+	+	-
RM TRIMCyp	-	-	-
PM TRIMCyp	-	-	-
CM TRIMCyp (DK)	+	-	-
CM TRIMCyp (NE)	-	-	-

*Summary of findings about interactions between each *TRIM5* allele and lentiviruses (Stremlau et al., 2004; Newman et al., 2006; Liao et al., 2007; Brennan et al., 2008; Virgen et al., 2008; Dietrich et al., 2011; Saito et al., 2012b). “+” denotes restrictive, while “-” denotes not restrictive against each lentivirus, respectively.*

## CONCLUSIONS AND FUTURE DIRECTIONS

Most HIV-1mts were constructed with the aim of evading from TRIM5 and APOBEC3-mediated restriction. In the future research, as discussed above, we should also focus on other factors such as BST-2 and SAMHD1. It will be promising to modify viral genome in order to overcome these restrictions. We expect that such procedure will lead to the construction of a new HIV-1mt with the ability to infect various macaques persistently.

Also, as discussed in the “History of HIV-1 animal models” chapter, an R5-tropic virus would be promising to reproduce the transmission, latency, and pathogenicity of HIV-1 in macaques. In the future study, the construction of an R5-tropic virus on the HIV-1mt backbone would encourage us to examine the antiviral agents, vaccines, and microbicides in macaques. Moreover, HIV-1mt that robustly replicate and induce pathogenicity in monkeys will make feasible to investigate the role and mechanism of HIV-1 accessory genes in the HIV-1 lifecycle, persistence, and pathogenesis. In summary, although the road to the containment of HIV-1 epidemic may be long and steep, we have been moving forward slowly but steadily.

## Conflict of Interest Statement

The authors declare that the research was conducted in the absence of any commercial or financial relationships that could be construed as a potential conflict of interest.
